# Detection of brain lesions after catheter ablation depends on imaging criteria: insights from AXAFA-AFNET 5 trial

**DOI:** 10.1093/europace/euad323

**Published:** 2023-10-28

**Authors:** Karl Georg Haeusler, Felizitas A Eichner, Peter U Heuschmann, Jochen B Fiebach, Tobias Engelhorn, David Callans, Tom De Potter, Philippe Debruyne, Daniel Scherr, Gerhard Hindricks, Hussein R Al-Khalidi, Lluis Mont, Won Yong Kim, Jonathan P Piccini, Ulrich Schotten, Sakis Themistoclakis, Luigi Di Biase, Paulus Kirchhof

**Affiliations:** Atrial Fibrillation NETwork association (AFNET), Mendelstr. 11, 48149 Münster, Germany; Department of Neurology, Universitätsklinikum Würzburg Josef-Schneider-Str. 11, 97080 Würzburg, Germany; Institute of Clinical Epidemiology and Biometry, University Würzburg, Würzburg, Germany; Institute of Clinical Epidemiology and Biometry, University Würzburg, Würzburg, Germany; Clinical Trial Center, University Hospital Würzburg, Würzburg, Germany; Institute of Medical Data Science, University Hospital Würzburg, Würzburg, Germany; Center for Stroke Research Berlin, Charité—Universitätsmedizin Berlin, Berlin, Germany; Department of Neuroradiology, University of Erlangen-Nuremberg, Erlangen, Germany; Hospital of the University of Pennsylvania, Philadelphia, USA; Cardiovascular Center, OLV Hospital, Aalst, Belgium; Hospital Imelda, Bonheiden, Belgium; Division of Cardiology, Medical University Graz, Austria; Deutsches Herzzentrum der Charité, Berlin, Germany; Department of Biostatistics & Bioinformatics, Duke University School of Medicine, Durham, NC, USA; Hospital Clínic, Universitat de Barcelona, IDIBAPS, Barcelona, Spain; Department of Cardiology, Aarhus University Hospital, Aarhus, Denmark; Duke Clinical Research Institute (DCRI), Durham, NC, USA; Division of Cardiology Duke University Medical Center, Duke University, Durham NC, USA; Atrial Fibrillation NETwork association (AFNET), Mendelstr. 11, 48149 Münster, Germany; Departments of Cardiology and Physiology, University Maastricht, Maastricht, The Netherlands; Department of Cardiology, Ospedale dell’Angelo, Mestre-Venice, Italy; Albert Einstein College of Medicine at Montefiore Hospital, New York, NY, USA; Texas Cardiac Arrhythmia Institute at St.David’s Medical Center, Austin, TX, USA; Atrial Fibrillation NETwork association (AFNET), Mendelstr. 11, 48149 Münster, Germany; University of Birmingham Institute of Cardiovascular Sciences, Birmingham, UK; Department of Cardiology, University Heart and Vascular Center UKE Hamburg, Hamburg, Germany; German Center for Cardiovascular Research, Partner site Hamburg/Kiel/Lübeck, Germany

**Keywords:** Ablation, Diffusion-weighted imaging, Slice thickness, Field strengths, Brain MRI

## Abstract

**Aims:**

Left atrial catheter ablation is well established in patients with symptomatic atrial fibrillation (AF) but associated with risk of embolism to the brain. The present analysis aims to assess the impact of diffusion-weighted imaging (DWI) slice thickness on the rate of magnetic resonance imaging (MRI)–detected ischaemic brain lesions after ablation.

**Methods and results:**

AXAFA-AFNET 5 trial (NCT02227550) participants underwent MRI using high-resolution (hr) DWI (slice thickness: 2.5–3 mm) and standard DWI (slice thickness: 5–6 mm) within 3–48 h after ablation. In 321 patients with analysable brain MRI (mean age 64 years, 33% female, median CHA_2_DS_2_-VASc 2), hrDWI detected at least one acute brain lesion in 84 (26.2%) patients and standard DWI in 60 (18.7%; *P* < 0.01) patients. High-resolution diffusion-weighted imaging detected more lesions compared to standard DWI (165 vs. 104; *P* < 0.01). The degree of agreement for lesion confirmation using hrDWI vs. standard DWI was substantial (*κ* = 0769). Comparing the proportion of DWI-detected lesions, lesion distribution, and total lesion volume per patient, there was no difference in the cohort of participants undergoing MRI at 1.5 T (*n* = 52) vs. 3 T (*n* = 269).

**Conclusion:**

The pre-specified AXAFA-AFNET 5 sub-analysis revealed significantly increased rates of MRI-detected acute brain lesions using hrDWI instead of standard DWI in AF patients undergoing ablation. In comparison to DWI slice thickness, MRI field strength had a no significant impact in the trial. Comparing the varying rates of ablation-related MRI-detected brain lesions across previous studies has to consider these technical parameters. Future studies should use hrDWI, as feasibility was demonstrated in the multicentre AXAFA-AFNET 5 trial.

## Graphical Abstract

**Graphical Abstract:**
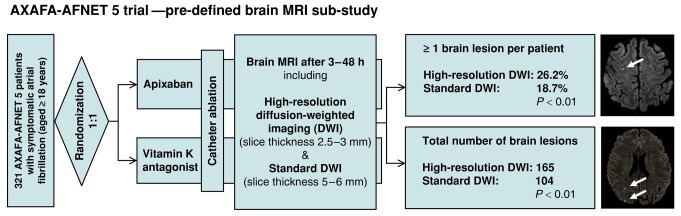
Design of the pre-defined brain magnetic resonance imaging (MRI) sub-study of the AXAFA-AFNET 5 trial also demonstrating the main results comparing MRI-detected acute ischaemic brain lesions using high-resolution vs. standard diffusion-weighted imaging (DWI).

**What’s new?**

- While left atrial catheter ablation with symptomatic atrial fibrillation (AF) is associated with brain magnetic resonance imaging (MRI)–detected acute brain lesions in 10–40% of all patients, depending on procedure-related factors, the investigator-initiated, multicentre AXAFA-AFNET 5 trial is the first randomized trial including a pre-defined MRI sub-study using standard diffusion-weighted imaging (DWI; slice thickness 5–6 mm) in addition to high-resolution DWI (slice thickness 2.5–3 mm).
- Compared to standard DWI, high-resolution DWI significantly increased the rate of MRI-detected acute brain lesions after ablation per patient as well as the total number of lesions, while MRI field strength had a no significant impact in the trial.
- Technical details of brain MRI and especially DWI slice thickness must be considered by comparing reported rates of ablation-related brain lesions of previous studies.
- Future interventional studies should use high-resolution DWI to detect acute brain lesions, as feasibility was demonstrated in the AXAFA-AFNET 5 trial.

## Introduction

Atrial fibrillation (AF) is the most prevalent cardiac arrhythmia worldwide. Left atrial catheter ablation is an increasingly employed treatment option in patients with symptomatic AF to improve AF-related symptoms and quality of life.^1–3^ Catheter ablation is a main component of rhythm control therapy, which has the potential to reduce cardiovascular endpoints in patients with AF diagnosed within 12 months.^4^ However, left atrial catheter ablation comes at the cost of a measurable peri-procedural risk of embolism to the brain causing ischaemic stroke in <0.5% of ablated patients.^2,5^ Brain magnetic resonance imaging (MRI) using diffusion-weighted imaging (DWI) identifies clinically unapparent, so-called ‘silent’ or ‘covert’ acute brain lesions in 10–40% of all patients undergoing left atrial catheter ablation, depending on procedure-related factors, e.g. ablation catheter used, procedure duration, ablation method, peri-procedural anticoagulation on top of heparinization, and number of cardioversions.^6–11^ In addition, MRI parameters like DWI slice thickness^12^ or MRI field strengths may affect the detection of ablation-related brain lesions, which may add to cognitive decline in the long term.^13–15^ As demonstrated in (non-ablated) patients with acute ischaemic stroke, high-resolution DWI (hrDWI) using a DWI slice thickness of ≤3 mm (instead of ≥5 mm in routine care) increased spatial resolution, increased the signal to noise, and decreased artefacts leading to improved lesion detection, predominately in the cortical grey matter.^16,17^

To clarify the impact of DWI slice thickness on the detection rate of acute ischaemic brain lesions after catheter ablation, patients enrolled to the investigator-initiated, multicentre, randomized *Anticoagulation using the direct factor Xa inhibitor apixaban during Atrial Fibrillation catheter Ablation: Comparison to vitamin K antagonist therapy* (AXAFA-AFNET 5) MRI sub-study underwent standard DWI (using a slice thickness of 5–6 mm) in addition to hrDWI (using a slice thickness of 2.5–3 mm).^18^ The investigator-initiated AXAFA-AFNET 5 trial demonstrated that peri-procedural anticoagulation using apixaban is a safe alternative to vitamin K antagonists for patients with symptomatic paroxysmal AF undergoing catheter ablation with regard to major bleeding, stroke, and cognitive function.^5^ The pre-defined analysis of available brain MRIs revealed no effect of random treatment on acute brain lesions or cognitive function at 3 months after ablation.^5,6^

## Methods

### Study design and study population

The prospective, parallel-group, 1:1 randomized, open AXAFA-AFNET 5 trial was conducted in 49 centres in eight European countries and the USA in accordance with the Declaration of Helsinki and the International Conference on Harmonization Good Clinical Practice Guidelines.^18^ The ethical review board of all study centres approved the study protocol. All patients provided written informed consent. It was not appropriate to involve patients or the public in the design, conduct, reporting, or dissemination plans of our research. Inclusion and exclusion criteria were published previously and are listed in the Supplementary material online, *Table S1*. The study enrolled 674 patients with symptomatic non-valvular AF scheduled for a first ablation.^5^ Patients randomized to apixaban received 5 mg twice daily pre-ablation, which was continued during the ablation procedure without interruption. Dose adjustment was done according to its label. Patients randomized to a vitamin K antagonist (VKA) were treated according to site-specific anticoagulation therapy routine aiming for a target international normalizied ratio of 2–3.^18^ The ablation procedure could be either radiofrequency ablation or cryoballoon ablation and was conducted according to local practice. The trial sponsor was executed by the Clinical Research Institute, Munich, Germany, and sponsored by the AFNET, Münster, Germany. Brain MRIs were conducted in 25 centres in eight European countries and the USA using 1.5 or 3 T within 3–48 h after the ablation procedure. Offering participation to all eligible patients at MRI sites, 333 study patients underwent brain MRI, of which 12 scans were unanalysable (see Supplementary material online, *Figure S1* for details).^5,6^ Baseline characteristics, demographics, and ablation characteristics of the brain MRI sub-study population were similar compared to the total AXAFA study population, and the AXAFA study population at study sites is able to provide brain MRI.^6^

### Brain magnetic resonance imaging

The AXAFA-AFNET 5 imaging charts (see Supplementary material online, *Table S2* for details) included hrDWI with 2.5–3 mm slice thickness as well as standard DWI with 5–6 mm slice thickness in the same patient to assess acute ischaemic brain lesions and to compare the impact of hrDWI vs. standard DWI.^18^ The additional acquisition time for hrDWI varied between 30 and 90 s across study centres. Magnetic resonance images were centrally read for new brain lesions in a core lab (Neuroscios, St Radegund, Graz, Austria). Two expert neuroradiologists (J.B.F, T.E.) served as independent raters and were blinded to randomization, MRI field strengths, DWI slice thickness used, and clinical information. The number of brain lesions and the total volume of brain lesions were documented per patient. The presence and localization of acute brain lesions was documented according to brain-supplying arteries. The volume of every single brain lesion was assessed by using DWI (at *b* = 1000 s/mm^2^) for planimetric delineation.

### Statistics

The analysis population included all randomized patients that underwent left atrial catheter ablation. Descriptive statistics for continuous variables were summarized as means [standard deviations (SDs)] or as medians (interquartile range (IQR): 25th, 75th percentiles) and counts (percentages) for categorical variables. Comparisons between continuous variables were performed with the two-sample *t*-test or Wilcoxon rank-sum test, depending on normality; comparisons between nominal variables were performed with Pearson’s *χ*^2^ test or Fisher’s exact test, as appropriate. All analyses were exploratory and tested two sided at the nominal significance level of 0.05. No adjustments were made for multiple testing. Cohen’s kappa was calculated to determine the agreement of lesion detection using hrDWI vs. standard DWI.^19^ All statistical analyses were performed using R version 3.4.3.

## Results

Baseline characteristics and ablation characteristics of 321 AXAFA-AFNET 5 patients with analysable MRI are depicted in *Table 1*. Mean age was 64 years, 33% were female, and the median CHA_2_DS_2_-VASc score was 2 points at the time of enrolment. Most patients underwent pulmonary vein isolation only, mainly using radiofrequency. Of the 321 patients, 52 (16.2%) underwent brain MRI at 1.5 T and 269 (83.8%) at 3 T (Supplementary material online, *Figure S1*). Apart from the type of energy used, ablation characteristics, patients’ baseline characteristics, and randomization to apixaban or VKA did not differ between the 1.5 T cohort and the 3 T cohort (*Table 1*). All 321 patients underwent standard DWI (slice thickness of 5–6 mm) and hrDWI (slice thickness of 2.5–3 mm) immediately afterwards.

**Table 1:** Baseline characteristics and demographics of AXAFA-AFNET 5 patients according to availability/field strengths of brain MRI

	Brain MRI 1.5/3 T *n* = 321	Brain MRI at 1.5 T *n* = 52	Brain MRI at 3 T *n* = 269	*P*-value
Age (years), median (IQR)	64 (58–70)	65 (58–71)	64 (58–69)	0.436
Female sex, *n* (%)	105 (33)	12 (23)	93 (35)	0.145
BMI (kg/m^2^), median (IQR)	28 (25–30)	28 (25–29)	28 (25–30)	0.847
Type of AF, *n* (%)				0.990
Paroxysmal	197 (61)	32 (62)	165 (61)	
Persistent	117 (36)	19 (37)	98 (36)	
Long-standing persistent	7 (2)	1 (2)	6 (2)	
CHA_2_DS_2_-VASc score, median (IQR)	2 (1–3)	2 (2–3)	2 (1–3)	0.221
Older than 75 years, *n* (%)	26 (8)	5 (10)	21 (8)	0.873
Prior stroke or TIA, *n* (%)	28 (9)	7 (13)	21 (8)	0.292
Hypertension, *n* (%)	295 (92)	50 (96)	245 (91)	0.342
Diabetes mellitus, *n* (%)	38 (12)	7 (13)	31 (12)	0.872
Symptomatic heart failure, *n* (%)	56 (17)	11 (21)	45 (17)	0.569
Vascular disease, *n* (%)	40 (12)	10 (19)	30 (11)	0.166
Carotid stenosis (>50%), *n* (%)	1 (0)	0 (0)	1 (0)	0.999
Anticoagulation before randomization, *n* (%)	41 (13)	8 (15)	33 (12)	0.697
Antiplatelets at randomization, *n* (%)	15 (5)	5 (10)	10 (4)	0.137
Statin before randomization, *n* (%)	111 (35)	22 (42)	89 (33)	0.262
Type of index catheter ablation, *n* (%)				0.059
Pulmonary vein isolation	286 (89)	47 (90)	239 (89)	
Pulmonary vein isolation + other	34 (11)	4 (8)	30 (11)	
Other	1 (0)	1 (2)	0 (0)	
Type of ablation energy, *n* (%)				<0.001
Radio frequency	217 (68)	50 (96)	167 (62)	
Cryoenergy	68 (21)	2 (4)	66 (825)	
Other	36 (11)	0 (0)	36 (13)	
Anticoagulation after randomization, *n* (%)				0.999
VKA	165 (50)	26 (50)	133 (49)	
Apixaban	167 (50)	26 (50)	136 (51)	
Use of TEE prior to ablation, *n* (%)	279 (87)	42 (81)	237 (88)	0.226
Randomization to ablation (days), median (IQR)	35 (22–46)	34 (22–44)	35 (21–47)	0.884
ACT during ablation (s), median (IQR)	325 (300–365)	339 (304–365)	320 (297–352)	0.44
Heparin dose during ablation (IU), median (IQR)	12 000 (10 000–15 983)	10 863 (10 000–15 188)	12 000 (9600–16 000)	0.783
Cardioversion(s) during ablation, median (IQR)	0 (0–1)	0 (0–1)	0 (0–1)	0.813

ACT, activated clotting time; BMI, body mass index; TEE, transesophageal echocardiogram; TIA, transient ischemic attack; VKA, vitamin K antagonist.

**Figure 1:**
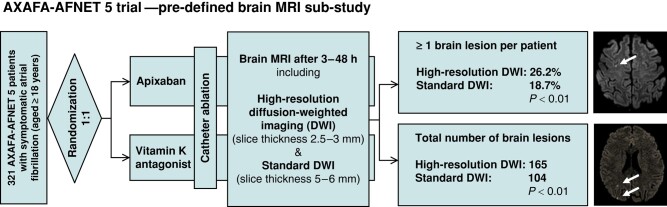
Design of the pre-defined brain MRI sub-study of the AXAFA-AFNET 5 trial also demonstrating the main results comparing MRI-detected acute ischaemic brain lesions using hr vs. standard DWI. DWI, diffusion-weighted imaging; MRI, magnetic resonance imaging.

Using standard DWI, 104 acute brain lesions were found in 60 (18.7%) patients, whereas hrDWI revealed 165 acute brain lesions in 84 (26.2%) patients (*Figure 1* and *Table 2*). Compared to standard DWI, hrDWI detected a significantly higher number of lesions (*P* < 0.01) in a significantly higher number of patients with at least a single lesion (*P* < 0.01). Examples for hrDWI and standard DWI in single patients are depicted in Supplementary material online, *Figure S2*. The distribution of lesion volumes per patients differed, as hrDWI more frequently detected patients with a total lesion volume ≤50 mm^3^.

**Table 2:** Brain lesions detected by hrDWI compared to standard DWI in 321 patients with analysable MRI

	hrDWI *n* = 321	DWI *n* = 321	*P*-value
Total number of brain lesions detected	165	104	<0.01
Volume of brain lesions detected per patient			<0.01
Total volume <20 mm^3^	9 (3)	2 (1)	
Volume 21–50 mm^3^	26 (8)	13 (4)	
Volume 51–100 mm^3^	22 (7)	19 (6)	
Volume >100 mm^3^	27 (8)	26 (8)	
Patients with at least one detected lesion, *n* (%)	84 (26)	60 (19)	<0.01
DWI-detected brain lesions and MRI field strengths, *n*			
1.5 Tesla (in 52 patients)	26	11	0.006
3 Tesla (in 269 patients)	139	93	<0.01

In 52 patients undergoing 1.5 T MRI, standard DWI detected 11 acute brain lesions in 8 (15.3%) patients, and hrDWI detected 26 acute brain lesions in 16 (30.8%) patients (*P* = 0.006; *Table 2*; Supplementary material online, *Figure S2*). In 268 patients undergoing 3 T MRI, standard DWI detected 93 acute brain lesions in 52 (19.4%) patients, and hrDWI detected 139 acute brain lesions in 68 (28.6%) patients (*P* < 0.01; *Table 3*; Supplementary material online, *Figure S2*). The number of detected lesions per patient and total lesion volume according to standard DWI and hrDWI is shown in *Table 3*. There was no significant difference in the 1.5 T cohort vs. the 3 T cohort using standard DWI or hrDWI regarding the proportion of detected lesions per patient (see Supplementary material online, *Figure S2*) and total lesion volume. Furthermore, the distribution of affected vessel territories was similar in the 1.5 T cohort and the 3 T cohort.

**Table 3:** Acute ischaemic brain lesions detected in 321 patients with analysable MRI according to field strength

	Brain MRI at 1.5 T *n* = 52	Brain MRI at 3 T *n* = 269	*P*-value
Standard DWI			
Detected lesions, *n* (%)	8 (15)	52 (19)	0.636
Number of lesion per patient, *n* (%)			0.808
0	44 (85)	217 (81)	
1	6 (12)	30 (11)	
2	1 (2)	14 (5)	
≥3	1 (2)	7 (3)	
Total lesion volume, *n* (%)			0.774
<20 mm^3^	0 (0)	2 (1)	
≥20 and <50 mm^3^	3 (6)	10 (4)	
≥50 and <100 mm^3^	2 (4)	17 (6)	
≥100 mm^3^	3 (6)	23 (9)	
high-resolution DWI			
Detected lesions, *n* (%)	16 (31)	68 (25)	0.514
Number of lesion per patient, *n* (%)			0.718
0	36 (69)	201 (75)	
1	11 (21)	35 (13)	
2	3 (6)	18 (7)	
≥3	2 (4)	15 (6)	
Total lesion volume, *n* (%)			0.943
<20 mm^3^	2 (4)	7 (3)	
≥20 and <50 mm^3^	5 (10)	21 (8)	
≥50 and <100 mm^3^	4 (8)	18 (7)	
≥100 mm^3^	5 (10)	22 (8)	
Affected vessel territory, *n* (%)			
*Arteria cerebri media*	11 (21)	51 (19)	0.861
*Arteria cerebri* anterior	0 (0)	8 (3)	0.439
*Arteria cerebri* posterior	3 (6)	17 (6)	0.999
Vertebral/basilar artery	5 (10)	15 (6)	0.430

DWI, diffusion-weighted imaging.

The degree of agreement for lesion detection using hrDWI vs. standard DWI was substantial (*κ* = 0.769) in all 321 patients, as well as in 52 patients undergoing brain MRI at 1.5 T (*κ* = 0.695) and in 269 patients undergoing brain MRI at 3 T (*κ* = 0.808).

## Discussion

To the best of our knowledge, this is the largest prospective brain MRI study comparing standard DWI to hrDWI in AF patients undergoing left atrial catheter ablation.^5,6^ Using a multicentre approach, we demonstrate that brain MRI using hrDWI significantly improves the detection of ablation-related acute brain lesions, if compared to standard DWI. In particular, hrDWI detected at least one acute brain lesion in a significantly higher proportion of study patients (26% vs. 19% using standard DWI) and a significantly higher number of lesions (*n* = 165 vs. *n* = 104 using standard DWI) throughout the brain. In addition, hrDWI in comparison to standard DWI more frequently detected a total lesion volume ≤50 mm^3^. A small single-centre study from China reported similar findings regarding lesion load in patients with ablation-related brain lesions by comparing DWI using 1 mm vs. 5 mm slice thickness at 3 T in 55 AF patients,^12^ despite the fact that a slice thickness of 1 mm is not established in clinical practice.

Of note, the significantly higher rate of hrDWI vs. standard DWI detected lesions in AXAFA-AFNET 5 was similar using 1.5 T MRI compared to 3 T MRI, which is another novel finding of our analysis. Our finding is in line with a previous meta-analysis in patients with transient global amnesia (TGA),^20^ demonstrating a low impact of field strengths, despite a higher contrast-to-noise ratio in high-field-strength MRI. Subsequently, one might argue that 1.5 and 3 T MRI has equivalent sensitivity for detecting ablation-related brain lesions, if hrDWI is performed.

Taken together, our findings help to explain the varying frequency of MRI-detected acute brain lesions post-ablation in previous cohorts using similar ablation catheters but different DWI parameters while most often using a DWI slice thickness of 5–6 mm.^2,7–9,12^

The pre-defined analysis of the AXAFA-AFNET 5 study has several strengths beside the rather large sample size and the well-standardized assessment.^18^ It has to be mentioned, in addition, that independent raters were blinded to clinical or MRI information. Despite the fact that many of the contributing MRI teams in this multicentre study do mainly or exclusively cardiac MRI exams in their daily practice, the technical quality of the study-related brain exams was excellent, and the degree of agreement for lesion detection using hrDWI vs. standard DWI was substantial. In conclusion, hrDWI is feasible in a phase II trial setup and should be regarded as standard for future trials, which may help to further optimize the safety of the ablation procedure by reducing the procedure-related embolic risk. Besides the use of specific protection devices and optimization of peri-procedural anticoagulation, the types of energy application in the left atrium are modifiable drivers of ablation-associated stroke risk.^14,21^

Despite the reported strength, the following limitations must be considered. Although this was a pre-specified analysis of AXAFA-AFNET 5 trial,^18^ the brain MRI sub-study was not specifically powered to detect differences of brain lesions with regard to DWI slice thickness or MRI field strength. About half of the total AXAFA-AFNET 5 cohort underwent brain MRI. However, the participation rate is rather high if compared to the brain MRI sub-study of the randomized ELIMINATE-AF trial, including 177 (28%) of 632 AF patients only.^2^ Conducting a single brain MRI within 3–48 h post-ablation and no MRI immediately before ablation, we cannot rule out acute brain lesions within days before the ablation procedure, as DWI lesions are present for up to 14 days.^7^ Moreover, study patients were not examined at 1.5 and 3 T, as this was not feasible in the vast majority of study sites. Despite the fact that neuroradiologists were also blinded to information on slice thickness, the number of available slices differed in hrDWI and standard DWI data sets. Finally, we were not able to assess the impact of other technical MRI parameters like number of active receiver channels in the head coil or vendor-specific sequence parameters.

## Conclusions

Using hrDWI instead of standard DWI, this pre-specified analysis of the AXAFA-AFNET 5 trial revealed markedly increased rates of MRI-detected acute brain lesions in patients with symptomatic AF undergoing first-time catheter ablation. In comparison to DWI slice thickness, MRI field strength had no significant impact in the trial. Comparing the varying rates of ablation-related brain lesions across previous studies must consider the technical details revealed by the pre-specified analysis of the AXAFA-AFNET 5 trial. Future interventional studies focusing on reducing the risk of ablation-related embolization to the brain should use hrDWI to detect acute lesions, as feasibility of this approach was demonstrated in the multicentre AXAFA-AFNET 5 trial.

## Supplementary Material

euad323_Supplementary_Data

## Data Availability

The data will be shared upon reasonable request to the AXAFA-AFNET 5 trial sponsor (via axafa@af-net.eu).
