# Supplementary material for: Detection of brain lesions after catheter ablation depends on imaging criteria: insights from AXAFA-AFNET 5 trial
Source: Europace. 2023 Oct 28;25(12):euad323. doi: 10.1093/europace/euad323 (PMC10963060; doi:10.1093/europace/euad323)
Supplement: euad323_Supplementary_Data [file euad323_supplementary_data.docx]

**ONLINE SUPPLEMENT**

**Figure S1** CONSORT flow chart of the AXAFA-AFNET 5 brain MRI sub-study.

Number of subjects randomized to AXAFA
(n=674)

Number of subjects who underwent first-time ablation
(n=634)

Randomized to VKA
(n=315)

Randomized to Apixaban

(n=319)

Withdrawal

(n=2)

No. of subjects undergoing

MRI at 1.5 Tesla (n=26) &

MRI at 3 Tesla (n=133)

3-months follow-up visit

(n=159)

No. of subjects undergoing

MRI at 1.5 Tesla (n=26) &

MRI at 3 Tesla (n=136)

No. of subjects receiving brain MRI

(n=165)

No. of subjects receiving brain MRI

(n=168)

No. of subjects included in

ITT analysis undergoing standard & high-resolution DWI

(n=159)

No. of subjects included in

ITT analysis undergoing standard & high-resolution DWI (n=162)

Withdrawal

(n=1)

Withdrawal before ablation

(n=40)

Unanalyzable

brain MRI

(n=6)

Unanalyzable

brain MRI

(n=6)

**Figure S2** Acute brain lesions (indicated by arrows) detected by 1.5 Tesla MRI (1&2) or 3 Tesla MRI (3&4) within 3-48 hours after left atrial catheter ablation using high-resolution DWI (A) or standard DWI (B) in four study patients (1-4).


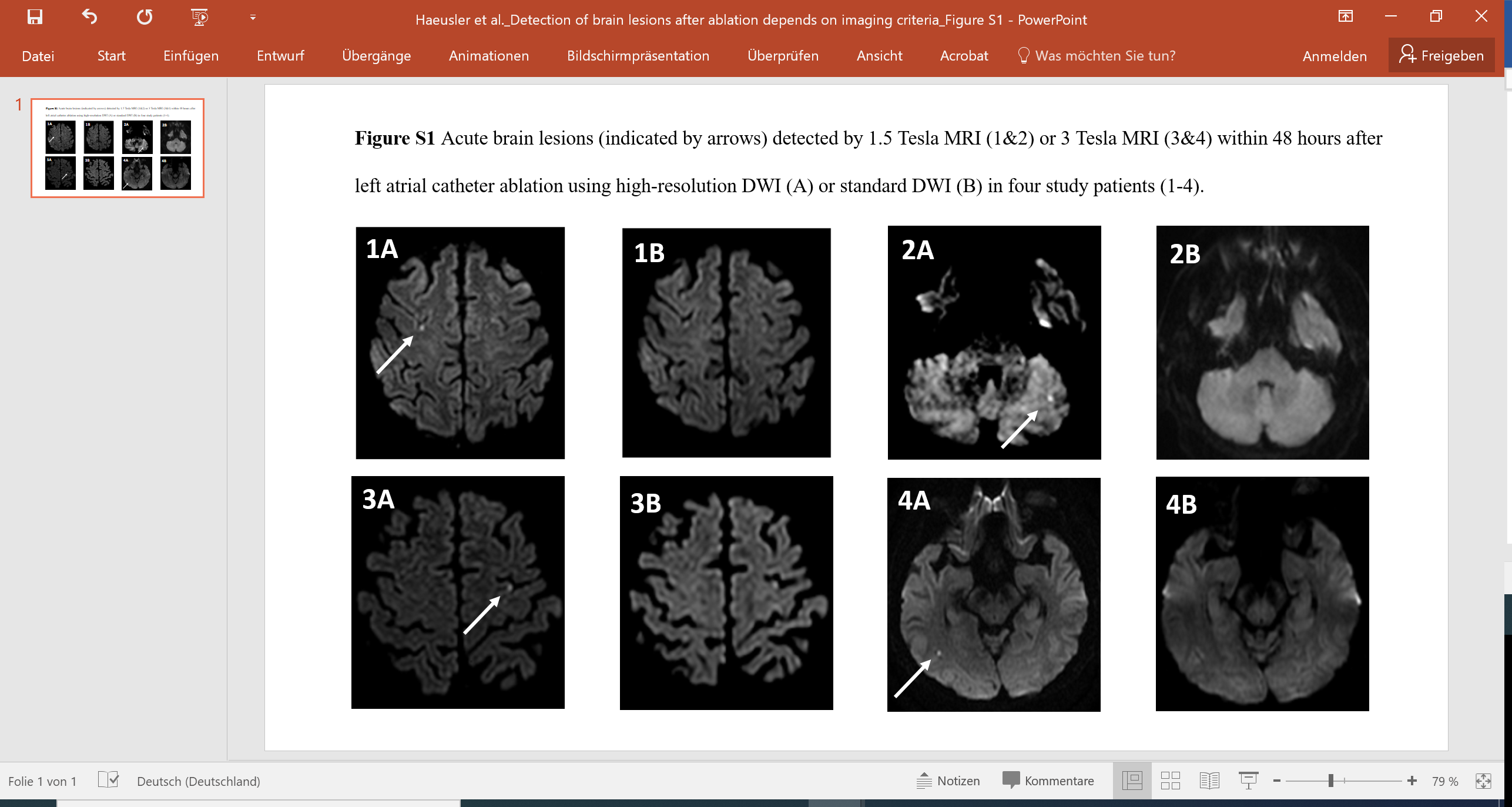


**Table S1** AXAFA-AFNET 5 inclusion and exclusion criteria [4].

| **Inclusion criteria** | **Exclusion criteria** |
| --- | --- |
| Non-valvular atrial fibrillation (ECG-documented) with a clinical indication for catheter ablation | Any disease that limits life expectancy to less than 1 year |
| Clinical indication to undergo catheter ablation on continuous anticoagulant therapy | Participation in another clinical trial, either within the past 2 months or still ongoing |
| Provision of signed informed consent | Previous participation in AXAFA |
| Age ≥ 18 years | Pregnant women or women of childbearing potential not on adequate birth control: only women with a highly effective method of contraception (oral contraception or intra-uterine device) or sterile women can be randomized |
| Presence of at least one of the CHADS_2_ stroke risk factors (Stroke or TIA, age ≥ 75 years, hypertension, defined as chronic treatment for hypertension, estimated need for continuous antihypertensive therapy or resting blood pressure > 145/90 mm Hg, diabetes mellitus, symptomatic heart failure (NYHA ≥ II). | Breastfeeding women |
|  | Drug abuse or clinically manifest alcohol abuse |
|  | Any stroke within 14 days before randomization |
|  | Concomitant treatment with drugs that are strong dual inhibitors of cytochrome P450 3A4 (CYP3A4) and P‑glycoprotein (P-gp) or strong dual inducers of CYP3A4 and P-gp |
|  | Valvular AF (as defined by the focused update of the ESC guidelines on AF, i.e. severe mitral valve stenosis, mechanical heart valve). Furthermore, patients who underwent mitral valve repair are not eligible for AXAFA |
|  | Any previous ablation or surgical therapy for AF |
|  | Cardiac ablation therapy for any indication (catheter-based or surgical) within 3 months prior to randomization |
|  | Clinical need for “triple therapy” (combination therapy of clopidogrel, acetylsalicylic acid, and oral anticoagulation) |
|  | Other contraindications for use of VKA or apixaban |
|  | Documented atrial thrombi less than 3 months prior to randomization |
|  | Severe chronic kidney disease with an estimated glomerular filtration rate (GFR) < 15 ml/min |

**Table S2** Imaging criteria for acute brain magnetic resonance imaging [4].

| **Sequence** | **Sequence type** | **Repetition time** | **Echo time** | **Averages**  **b-values** | **Field-of-view** | **Acquisition**  **matrix** | **Slice thickness** | **Gap** | **Slices coverage** |
| --- | --- | --- | --- | --- | --- | --- | --- | --- | --- |
| Standard diffusion-weighted imaging | Spin echo -Echo planar imaging | Local standard | Local standard | b = 0  b = 1000 | Local standard | Local standard | 5 - 6 mm | 0 | 20 - 25/  whole brain |
| High resolution diffusion-weighted imaging or diffusion tensor imaging | Spin echo -Echo planar imaging | > 8000 | 1.5 Tesla:  < 120  3.0 Tesla:  < 100 | b = 0  1-2 Averages  b = 1000  6 Averages | 220 - 300 mm | 1.5 Tesla:  ≥ 128x128  3.0 Tesla:  ≥ 192x128 | 2.5 - 3 mm | 0 | 40-50/  whole brain |
| Fluid attenuated inversion recovery | Inversion recovery | > 8000  Inversion time: scanner depended |  | 1 | 220 - 300 mm | 1.5 Tesla:  ≥ 192x128  3.0 Tesla:  ≥ 256x192 | 5 - 6 mm | max 10% | 20-25/  whole brain |
| T2*, Susceptibility weighted imaging or the like | Gradient echo | 620 | 20 | 1 | 220 - 300 mm | 1.5 Tesla:  ≥ 192x128  3.0 Tesla:  ≥ 256x192 | 5 - 6 mm | max 10% | 20-25/  whole brain |
